# Medical School Cohorts and Preparedness to Work With Individuals From Different Backgrounds: A Cross‐Sectional Study

**DOI:** 10.1002/hsr2.72563

**Published:** 2026-05-31

**Authors:** Douglas Grbic, Hershel Alexander, Dorothy A. Andriole, Mytien Nguyen, Charity Miller, Geoffrey Young

**Affiliations:** ^1^ Association of American Medical Colleges Washington DC USA; ^2^ Yale University School of Medicine New Haven Connecticut USA

## Abstract

**Background and Aims:**

Medical schools seek to prepare students to work effectively with individuals from a variety of backgrounds. This study examined whether selected attributes of matriculants were associated with how graduates self‐assessed their skills to work with individuals from different backgrounds.

**Methods:**

This retrospective cross‐sectional study used data from 139,681 matriculants in 973 cohorts (2013‐2014 to 2019‐2020) at 139 U.S. MD‐degree medical schools, and who graduated through 2022‐2023. From the AAMC Graduation Questionnaire, we used the survey item, “The diversity within my medical school class enhanced my training and skills to work with individuals from different backgrounds,” to create the cohort‐level outcome measure Self‐Assessed Skills at Graduation (SASG). For our independent variable, we created a Gower distance‐based composite measure of within‐cohort variation based on selected socio‐demographic attributes (gender, race/ethnicity, and parental education), called Student Attribute Variation (SAV). Higher SAV values indicated greater heterogeneity in selected attributes.

**Results:**

SAV was positively correlated with SASG (r = 0.50, *p* < .001). Multilevel regression confirmed this association.

**Conclusion:**

Findings highlight that greater variation in student composition is related to higher perceived development of professional skills.

## 1 Introduction

To improve health for all, medical schools seek to prepare students to work effectively with individuals from a variety of backgrounds [1, 2]. Prior studies suggest that exposure to people from different backgrounds may influence medical students' professional development [3, 4]; however, empirical evidence linking cohort composition to perceived skill development remains limited. Using data from seven matriculating cohorts (2013–2014 to 2019–2020) who graduated through 2022–2023, we examined whether selected attributes of matriculants were associated with how graduates self‐assessed their skills to work with individuals from different backgrounds.

## 2 Methods

### 2.1 Study Sample and Data

We examined data from 139,681 matriculants at 139 medical schools who applied to MD‐granting medical schools through the Association of American Medical Colleges (AAMC) American Medical College Application System (AMCAS) [5], representing 95.1% (139,681/146,828) of matriculants nationally between 2013–2014 and 2019–2020.

Students who did not apply to medical school through AMCAS (e.g., Texas Medical and Dental Schools Application Service) were excluded (4255 [2.9%] of all 146,828), as were students (2121) from schools that did not have all seven matriculant cohorts (2121), students (542) who matriculated into either the University of Minnesota‐Duluth campus or the Joint Medical Program collaboration between the University of California's Berkeley and San Francisco campuses, students (115) at one school where most did not use AMCAS, and students (114) who transferred between schools. In total, 7147 students (4.9% of 146,828) were excluded, resulting in a final nonrandom sample of 139,681 students from 973 cohorts across 139 medical schools.

### 2.2 Measures

For each school's matriculating cohort, we created a Student Attribute Variation (SAV) composite metric of within‐cohort differences on selected demographic attributes. SAV represents the mean variation between pairs of students on three salient attributes: gender (consisting of man, woman, and no response/decline to answer), race/ethnicity (consisting of nine mutually exclusive categories: American Indian or Alaska Native; Asian; Black or African American; Hispanic or Latino; Native Hawaiian or Pacific Islander; White; some other race or ethnicity; multiple race/ethnicities; and missing race/ethnicity) and parental education (consisting of less than a bachelor's degree; bachelor's degree or higher, and missing). These attributes reflect established (but not exhaustive) dimensions of social background in higher education research.

For each cohort, the mean variation was calculated using Gower's distance [6]:

$$D_{\mathrm{ij}}=\sum_{k=1}^{v}d_{\mathrm{ijk}}/v,$$

where *D*_*ij*_ is the distance between students *i* and *j*, *v* is the number of attributes, and *d*_*ijk*_ is the distance between those students *i* and *j* for attribute *k*. Theoretically, a cohort's mean distance can range from 0 (all student pairs share the same values in all attributes) to 1 (no pairs share the same attributes). Therefore, the SAV reflects the mean distance between a random student and all others in the cohort.

Unlike many distance metrics for only numerical data, Gower distance provides a flexible approach to measuring similarity across variable types, yielding a school‐cohort‐level score, with higher values indicating greater heterogeneity in gender, race/ethnicity, and parental education.

We used an item on the AAMC Graduation Questionnaire [7] (completed voluntarily by 75%–80% of graduating students) to create the outcome measure Self‐Assessed Skills at Graduation (SASG), reflecting graduates' *perceived* preparedness to work with individuals from different backgrounds: *The diversity within my medical school class enhanced my training and skills to work with individuals from different backgrounds*. Responses ranged from 1 (*strongly disagree*) to 5 (*strongly agree*) [6]. This item takes a holistic approach to diversity, with the item not specifying how diversity should be defined.

### 2.3 Statistical Analysis

Bivariate correlation and a linear multilevel regression model (with cohorts clustered in medical schools) assessed the relationship between the cohort‐level SAV measure and the cohort‐level SASG measure. Statistical significance was set at 2‐sided *p* < 0.05. Data were analyzed using Stata 19 (StataCorp LLC). The AAMC Human Subjects Protection Program Office exempted the study from institutional board review.

## 3 Results

**Figure 1:**
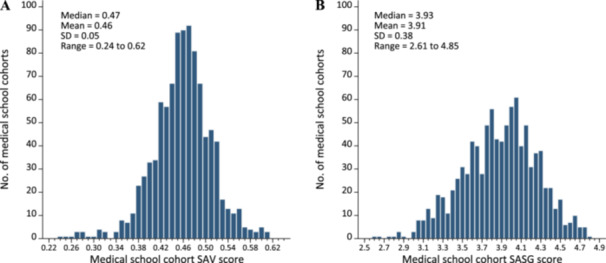
Distribution of medical school matriculant cohorts by mean Student Attribute Variation (SAV) score^a^ and mean Self‐Assessed Skills at Graduation (SASG) score^b^ for matriculating cohorts at graduation. *N* = 973. (A) Distribution of cohorts by SAV score. (B) Distribution of cohorts by SASG score. ^a^The SAV score was computed using the Gower distance formula, with the attributes gender (consisting of man, woman, and no response/decline to answer), race/ethnicity (consisting of nine mutually exclusive categories: American Indian or Alaska Native; Asian; Black or African American; Hispanic or Latino; Native Hawaiian or Pacific Islander; White; some other race or ethnicity; multiple race/ethnicities; and missing race/ethnicity) and parental education (consisting of less than a bachelor's degree; bachelor's degree or higher, and missing). Gender, race/ethnicity, and parental education were provided by medical school applicants when applying through the Association of American Medical Colleges (AAMC) American Medical College Application System (AMCAS). ^b^For each cohort, the mean SASG was computed from the AAMC Graduation Questionnaire item “The diversity within my medical school class enhanced my training and skills to work with individuals from different backgrounds.”

Across the 973 cohorts at 139 medical schools, the mean SAV was 0.46 (SD, 0.05), and the range was 0.24–0.62, as shown in Figure 1A. The mean SASG was 3.91 (SD, 0.38), and the range was 2.61–4.85, as shown in Figure 1B.

**Figure 2:**
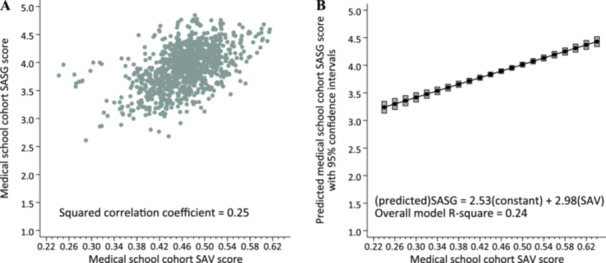
Association between the Student Attribute Variation (SAV) score^a^) and the Self‐Assessed Skills at Graduation (SASG) score^b^ for matriculating cohorts at graduation. *N* = 973. (A) Bivariate SAV–SASG correlation. (B) Predicted SASG score by SAV score.^c^ ^a^The SAV score was computed using the Gower distance formula, with the attributes gender (consisting of man, woman, and no response/decline to answer), race/ethnicity (consisting of nine mutually exclusive categories: American Indian or Alaska Native; Asian; Black or African American; Hispanic or Latino; Native Hawaiian or Pacific Islander; White; some other race or ethnicity; multiple race/ethnicities; and missing race/ethnicity) and parental education (consisting of less than a bachelor's degree; bachelor's degree or higher, and missing). Gender, race/ethnicity, and parental education were provided by medical school applicants when applying through the Association of American Medical Colleges (AAMC) American Medical College Application System (AMCAS). ^b^For each cohort, the mean SASG was computed from AAMC Graduation Questionnaire item “The diversity within my medical school class enhanced my training and skills to work with individuals from different backgrounds”. ^c^Predicted values of SASG were computed from a multilevel linear regression equation to account for the clustering of 973 cohorts in 139 medical schools.

Figure 2A plots each cohort by its mean SAV and mean SASG, showing a correlation between the two measures of 0.50 (*p* < 0.001) and indicating that 25% of the variance in SASG was accounted for by variation in SAV. For each mean SAV, Figure 2B shows the estimated mean SASG. The estimated multilevel regression coefficient was 2.98 (95% confidence interval, 2.51–3.44), indicating that for each 0.01 unit increase in SAV, SASG increased by 0.03 units (2.98 × 0.01).

## 4 Discussion

Our findings showed that greater within‐cohort SAV was associated with higher mean SASG. This finding aligns with prior research showing that learning environments with students from different backgrounds may contribute to students' professional development [3, 4]. This exploratory study introduced a novel metric of cohort‐level heterogeneity using selected student attributes available at matriculation, offering a feasible approach to analyzing how the composition of entering classes is associated with students' perceived readiness to professional practice.

The SAV measure was based on three salient indicators of social background—gender, race/ethnicity, and parental education. However, additional attributes of social background not included in this measure may increase exposure to different experiences, perspectives, and insights. Future studies could explore additional student characteristics such as age [3], college major [4], community college background [8], or other measures of formative experiences to expand this approach. Further, the current study did not examine institutional characteristics or curricular context [9, 10] that may shape students' perceptions or outcomes. Since roughly 75% of the variance in SASG remains unaccounted for, both cohort‐ and institution‐level characteristics warrant future investigations.

While our model accounted for between‐school variation, the limited number of cohorts (seven) per school constrained our ability to systematically assess whether the observed association between SAV and SASG is consistent across schools. Future studies with more longitudinal data could, therefore, explore this question using more flexible multilevel models that assess the extent to which this association might vary across schools.

This study has measurement limitations. The outcome relied on a single item from a voluntary survey, which may introduce self‐selection or response bias. The item reflects perceived preparedness rather than a measure of objective competence; however, this measure of graduates' self‐assessment is consistently available across institutions. Although the item offers a general indicator of students' self‐assessed readiness to work with individuals from different backgrounds, it does not specify which dimensions of background were most salient to graduating respondents. In addition, the exclusion of a small proportion of the students might have affected the representativeness of the study sample if these excluded students meaningfully differed in their characteristics. Finally, the findings are correlational and do not establish causation. However, future research might investigate how context‐level factors mediate the observed association.

## 5 Conclusions

Despite these limitations, our findings for seven recent cohorts extend prior research for much smaller cohorts that graduated 10–20 years ago [3, 4] by demonstrating that training with peers with a range of attributes is correlated with graduates' self‐assessed (not objectively measured) preparedness to work with individuals from different backgrounds, though these findings do not establish causal relationships. As medical education continues to evolve, understanding how student composition relates to professional skills development remains an important area of inquiry.

## Author Contributions

**Douglas Grbic:** conceptualization, methodology, software, formal analysis, data curation, supervision, resources, project administration, validation, visualization, writing – review and editing, writing – original draft, investigation. **Hershel Alexander:** conceptualization, investigation, writing – original draft, writing – review and editing, methodology, supervision, data curation. **Dorothy A. Andriole:** conceptualization, supervision, resources, writing – review and editing, writing – original draft, investigation, data curation. **Mytien Nguyen:** writing – original draft, conceptualization, methodology. **Charity Miller:** conceptualization, writing – original draft, methodology. **Geoffrey Young:** conceptualization, writing – review and editing, supervision.

## Funding

The authors have nothing to report.

## Ethics Statement

The AAMC human subjects office exempted this study from institutional board review because it did not constitute human participant research and used deidentified data.

## Conflicts of Interest

The authors declare no conflicts of interest.

## Transparency Statement

The lead author, Douglas Grbic, affirms that this manuscript is an honest, accurate, and transparent account of the study being reported; that no important aspects of the study have been omitted; and that any discrepancies from the study as planned (and, if relevant, registered) have been explained.

## Data Availability

Access to the data was granted to the authors solely for the purposes of this study. The data are proprietary and not publicly available. Requests for access can be submitted to the Association of American Medical Colleges via its Data Request form: https://www.aamc.org/request-aamc-data.
